# Multidomain Convolution Neural Network Models for Improved Event-Related Potential Classification

**DOI:** 10.3390/s23104656

**Published:** 2023-05-11

**Authors:** Xiaoqian Chen, Resh S. Gupta, Lalit Gupta

**Affiliations:** 1 School of Electrical, Computer, and Biomedical Engineering, Southern Illinois University, Carbondale, IL 62901, USA; xiaoqian.chen@siu.edu; 2 Center of Excellence for Stress and Mental Health, VA San Diego Healthcare System, San Diego, CA 92161, USA; resh.gupta@va.gov

**Keywords:** convolution neural networks, event-related potentials, continuous wavelet transform, scalograms, multidomain classifiers

## Abstract

Two convolution neural network (CNN) models are introduced to accurately classify event-related potentials (ERPs) by fusing frequency, time, and spatial domain information acquired from the continuous wavelet transform (CWT) of the ERPs recorded from multiple spatially distributed channels. The multidomain models fuse the multichannel Z-scalograms and the V-scalograms, which are generated from the standard CWT scalogram by zeroing-out and by discarding the inaccurate artifact coefficients that are outside the cone of influence (COI), respectively. In the first multidomain model, the input to the CNN is generated by fusing the Z-scalograms of the multichannel ERPs into a frequency-time-spatial cuboid. The input to the CNN in the second multidomain model is formed by fusing the frequency-time vectors of the V-scalograms of the multichannel ERPs into a frequency-time-spatial matrix. Experiments are designed to demonstrate (a) customized classification of ERPs, where the multidomain models are trained and tested with the ERPs of individual subjects for brain-computer interface (BCI)-type applications, and (b) group-based ERP classification, where the models are trained on the ERPs from a group of subjects and tested on single subjects not included in the training set for applications such as brain disorder classification. Results show that both multidomain models yield high classification accuracies for single trials and small-average ERPs with a small subset of top-ranked channels, and the multidomain fusion models consistently outperform the best unichannel classifiers.

## 1. Introduction

The accurate classification of event-related potentials (ERPs) is crucial in brain activity research and in brain-related clinical studies, evaluations, and diagnostics. Classification of ERPs can be improved by fusing frequency, time, and spatial domain information acquired from the scalograms of the continuous wavelet transforms (CWTs) of multichannel recordings of these signals. The frequency-time information is captured in the wavelet scalograms of each channel’s ERPs, and the spatial information is contributed from the spatially distributed channels across the scalp. Convolution neural network (CNN) classifiers are ideal for inputs presented in matrix and cuboid formats [1,2,3,4,5,6,7,8] and are, therefore, a good choice for processing single-channel matrix scalograms and multichannel scalograms combined into a cuboid. Furthermore, unlike traditional classifiers, which typically require extracting “hand-engineered” features from the three domains and selecting a set of features through trial and error, CNNs can be trained to extract intertwined frequency-time-spatial features directly. The shapes and dimensions of the filters in the convolution layers as well as the number of convolution layers offer flexibility in the type of multidomain intertwining desired. Classification models designed for single subjects, which is the primary focus of this study, can take two forms: customized classifier design for brain-computer interface (BCI)-type applications and group-based design for classifying brain activity and disorders in applications related to clinical studies, evaluations, and diagnostics. Customized classifiers are designed using the individual’s own ERPs, whereas group-based classifiers are typically trained on ERPs from a group of subjects having a similar disorder but tested on the ERPs of an individual not included in the training group. 

In a recent study related to the classification of unichannel (single channel) ERPs of single subjects [1], we introduced the Z-scalogram and the V-scalogram derived from the cone of influence (COI) of the standard scalogram of the CWT. The COI is a boundary that is superimposed on the wavelet scalogram to delineate the coefficients that are accurate from those that are inaccurate due to edge effects [1,9,10,11,12].

**Figure 1:**
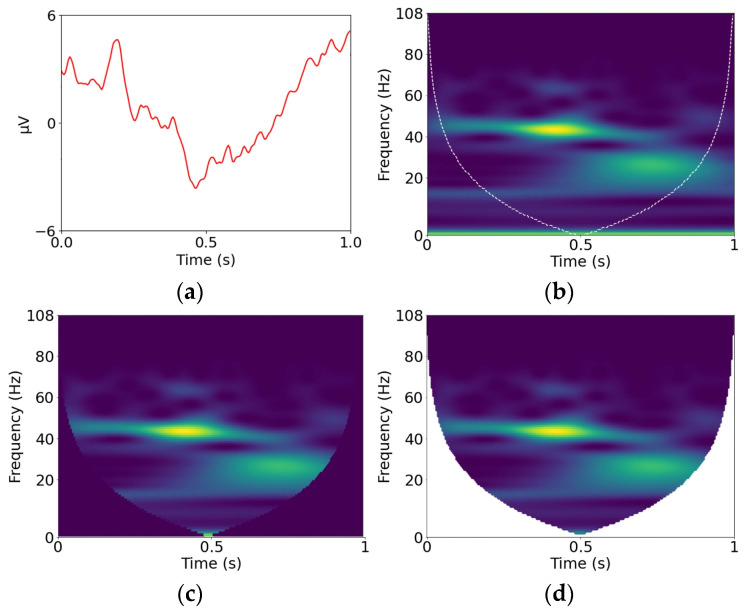
(**a**) ERP; (**b**) S-scalogram; (**c**) Z-scalogram; (**d**) V-scalogram.

The standard S-scalogram is obtained through “same” convolutions, the Z-scalogram is obtained from the S-scalogram by zeroing-out the inaccurate coefficients outside the COI, and the V-scalogram is obtained by completely cropping out the inaccurate coefficients outside the COI. The scalogram containing only the accurate coefficients corresponds to the scalogram that would be obtained through “valid” convolutions; therefore, it is referred to as the V-scalogram. Details of the various types of convolutions involved in defining the COI can be found in [1]. Figure 1, extracted from [1], shows the S-, Z-, and V-scalograms of an ERP. The S-scalogram shown in Figure 1b is the standard Morlet wavelet transform scalogram. In the study, feature vectors derived from the three scalograms were used to design support vector machines (SVMs), random forests (RFs), k-nearest neighbor (k-NN), multilayer perceptron neural networks (MLPs), and CNNs to classify synonymous and non-synonymous ERPs. It was shown that the Z-scalogram classifiers outperformed the S-scalogram classifiers, and the V-scalogram classifiers outperformed the S- and Z-scalogram classifiers. It was also shown that the CNN classifiers yielded the best performance. However, the study focused on customized classifier design for single subjects, did not involve group-based classification, and involved only unichannel classification with no attempt to fuse information from multiple channels.

In another recent study related to the classification of multiaxial human movement, various CNN models were developed to fuse “information” from multiple multiaxial sensors at the input-level (early fusion) and the output-level (late fusion) [13]. The information in input-level fusion is the input data or features extracted from the input data, whereas information in output-level fusion is the decisions of multiple classifiers or some measures at the outputs of the multiple classifiers [13,14,15,16,17,18,19]. In the multiaxial human movement classification study, it was shown that the fusion classifiers outperformed the uniaxial classifiers. However, the multiaxial temporal signals were used directly without any form of time-frequency transformations. Furthermore, the movement signals of all individuals were mixed prior to generating the training and test sets. That is, the experiments did not involve designing customized classifiers for each individual, nor did they involve designing group-based classifiers to test the movement signals of individuals not included in the training set.

The present study builds upon these studies and improves the classification of ERPs by using CNNs which fuse frequency, time, and spatial information acquired from the COI-modified scalograms of the multichannel ERPs. Two CNN-based multidomain classification models which fuse the COI-modified scalograms of multiple channels are introduced. In the first model, referred to as the Z-CuboidNet, the input to the CNN is a frequency-time-spatial cuboid formed by fusing the Z-scalograms of the multichannel ERPs, and in the second model, referred to as the V-MatrixNet, the CNN input is a frequency-time-spatial matrix formed by fusing the V-scalogram frequency-time vectors of the multichannel ERPs. CNNs are selected for the classifier component of the multidomain models because they are ideal for inputs presented in matrix and cuboid formats. Additionally, this study focuses on the design (training and testing) of both customized and group-based ERP classifiers. To the best of our knowledge, we are not aware of any other study that has been reported on the development of multidomain classification models similar to the Z-CuboidNet and V-MatrixNet. 

## 2. Materials and Methods

This section describes the following: (a) subsample averaging to facilitate the design of ERP classifiers, (b) formulations of the Z-CuboidNet and V-MatrixNet multidomain classifier models, (c) the formulations of the unichannel Z-MatrixNet and V-VectorNet models which are special cases of the multidomain models, (d) the ERP data set used to demonstrate the application and the performance of the classifier models, (e) cross-validation for group-based classifier design, (f) selection of subsets of channels for single subjects and the subjects combined into a group, (g) the Morlet wavelet transform, which, is the CWT used to generate the scalograms, and (h) the architectures, hyperparameters, and training options selected for the CNN models.

### 2.1. Subsample Averaging

An important issue related to ERP classification is the poor signal-to-noise ratio (SNR) of single trials; therefore, this issue is discussed before describing the formulations of the multidomain classification models. The SNR is improved for analysis and classification by averaging single trials from several repetitions of stimulus presentation [20,21,22]. The m-Subsample Averaging algorithm [1], which generates small-sample ERPs by repeated averaging of a small number of single trials drawn without replacement, can be employed to improve the performance over single trials. An ERP formed by averaging m single trials is referred to as an m-ERP. Subsample averaging enables the generation of a large ensemble of m-ERPs to facilitate classifier training. For consistency, single trials are referred to as 1-ERPs. The generalized formulations of the multidomain models in the following subsection assume m-ERPs. The experiments in this study are designed to evaluate the performances of the multidomain classifiers for m = 1, 2, and 4.

### 2.2. Multidomain Classifier Models

The most general formulations of the Z-CuboidNet and V-MatrixNet multidomain models involving multiple channels and polychotomous ERP classes are presented in this section. The channels are represented by d,d=0,1,…,(D−1), where, D is the number of selected channels, and the polychotomous classes are represented by $\omega_{j},j=1,2,\ldots ,\Omega ,$ where Ω is the number of classes. Using array (row-column) representations and zero-based indexing, a scalogram of an m-ERP is denoted by $G\left(f,t\right);\;f=0,1,...,\left(F-1\right),t=0,1,\ldots ,\left(T-1\right),$ where F is the number of frequency bands and T is the duration of the ERP. Furthermore, if an m-ERP in the ensemble of each channel is indexed by q,q=1,2,…,Q, where Q is the number of m-ERPs, the S-, Z-, and V-scalograms of the qth m-ERP of channel d belonging to class $\omega_{j}$ are represented by $G_{S,dj}^{\left(q\right)}\left(f,t\right)$, $G_{Z,dj}^{\left(q\right)}\left(f,t\right)$, and $G_{V,dj}^{\left(q\right)}\left(f,t\right)$, respectively. Classification involving unichannel and dichotomous classes are special cases of the general formulations.

Without loss of generality, the CNNs in the multidomain models consist of two convolution layers followed by a max pooling layer and a fully connected network (FCN). The activation functions in the convolution layers are ReLU and are tanh in the intermediate layers of the FCN. The output of the FCN is a softmax layer which has one output for each ERP class, and each output is interpreted as the posterior probability of the input class. The CNNs are trained with the gradient descent backpropagation algorithm using the cross-entropy loss function. The formulations of the 4 models, which follow next, describe the operations in the first convolution layer of each model in detail because the key differences between the models occur in this layer. The remaining operations are described briefly to point out additional differences and for the sake of completeness. For convenience, it will be assumed that all convolutions are “same convolutions” through zero-padding so that the filtered outputs have the same dimensions as the input to the convolution layer. The equations describe the cross-correlations of the input and filters in the convolution layers because the shifting, multiplication, and summing operations performed in CNNs are cross-correlation and not convolution. 

#### 2.2.1. Z-CuboidNet

The Z-CuboidNet fuses the Z-scalograms of the D channels into a frequency(f)-time(t)-spatial(d) cuboid given by

(1) $$\begin{array}{c} G_{Z,j}^{\left(q\right)}\left(f,t,d\right)={∆}_{d=0}^{\left(D-1\right)}G_{Z,dj}^{\left(q\right)}\left(f,t\right),\\ f=0,1,\ldots ,\left(F-1\right);\;t=0,1,\ldots ,\left(T-1\right);\;d=0,1,\ldots ,(D-1). \end{array}$$

where ∆ is the cuboid fusion operation. Note that $G_{Z,j}^{\left(q\right)}\left(f,t,d\right)$ is a cuboid generated by combining the Z-scalograms of the qth m-ERP of the D channels. The subscript j indicates that the Z-scalograms are generated from the class $\omega_{j}$ m-ERPs.

**Figure 2:**
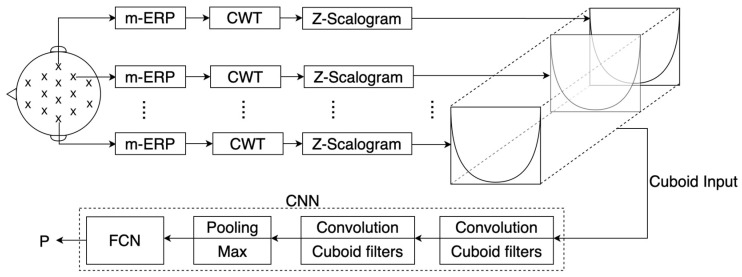
Block diagram of the Z-CuboidNet illustrating the formation of the fusion cuboid and the CNN which accepts the cuboid as the input.

The block diagram of the Z-CuboidNet is shown in Figure 2. In the first convolution layer, the cuboid input $G_{Z,j}^{\left(q\right)}\left(f,t,d\right)$ is convolved with $N_{1}$ cuboid filters $h^{\left[1,n\right]}\left(f,t,d\right),f=-\alpha ,\ldots ,0,\ldots ,\alpha ;\;t=-\beta ,\ldots 0,\ldots ,\beta ;\;$ $d=0,1,\ldots ,(D-1);\;n=1,2,\ldots ,N_{1}$, and the output of the $n^{th}$filter in the first convolution layer is given by

(2) $$\begin{array}{c} Y_{Z,j}^{\left(q\right)\left[1,n\right]}\left(f,t\right)=\sum_{d=0}^{\left(D-1\right)}\sum_{r=-\alpha }^{\alpha }\sum_{u=-\beta }^{\beta }h^{\left[1,n\right]}\left(r,u,d\right)G_{Z,j}^{\left(q\right)}\left(f+r,t+u,d\right),\\ f=0,1,\ldots ,\left(F-1\right),t=0,1,\ldots ,\left(T-1\right). \end{array}$$

It is important to note that cuboid filters are selected to extract intertwined frequency, time, and spatial features. Additionally interesting to note is that the result of convolving two cuboids is a matrix. After a bias is added to the filtered outputs and passed through the nonlinear ReLU activation function, the $N_{1}$ filtered outputs are combined into a $\left(F\times T\times N_{1}\right)$ cuboid feature map. The convolutions in the second layer are also selected to be cuboid convolutions to extract more complex cross-intertwined features across the cuboid feature map. The resulting matrices are combined into a cuboid after adding biases and passing through ReLU activations. The height and width of the cuboid is shrunk by the following max pooling layer. The max-pooled cuboid is flattened and presented as the input to the FCN. The output of the FCN is represented by the posterior class probability vector $P=(P_{\omega_{1}},P_{\omega_{2}},..,P_{\omega_{\Omega }})$, and the test input is assigned to a class using the maximum response rule. That is, the test input is assigned to class $\omega_{i}$ if

(3) $$P_{\omega_{i}}>P_{\omega_{j}}\;\mathrm{for}\;\mathrm{all}\;j\neq i.$$

#### 2.2.2. Z-MatrixNet

The Z-MatrixNet is a special case of the Z-CuboidNet for unichannel classification. The input cuboid reduces to a frequency-time matrix when D=1. A classifier is developed independently for the m-ERPs of each channel. This approach can be used to compare the performances of each channel classifier and to select the best unichannel classifier. Most importantly, the performances of the multidomain Z-CuboidNet classifier can be compared against the best unichannel Z-MatrixNet classifier. The input of channel d to the CNN is simply the Z-scalogram $G_{Z,dj}^{\left(q\right)}\left(f,t\right)$. In the first layer, the input matrix is convolved with $N_{1}$ matrix filters $h^{\left[1,n\right]}\left(f,t\right),f=-\alpha ,\ldots ,0,\ldots ,\alpha ;\;t=-\beta ,\ldots ,0,\ldots ,\beta ,n=1,2,\ldots ,N_{1}$. The output of filter n is given by the matrix convolution,

(4) $$\begin{array}{c} Y_{Z,dj}^{\left(q\right)\left[1,n\right]}\left(f,t\right)=\sum_{r=-\alpha }^{\alpha }\sum_{u=-\beta }^{\beta }h^{\left[1,n\right]}\left(r,u\right)G_{Z,dj}^{\left(q\right)}\left(f+r,t+u\right),\\ f=0,1,\ldots ,\left(F-1\right);\;t=0,1,\ldots ,\left(T-1\right). \end{array}$$

The matrix filters extract features that are coupled across frequency and time. Note that convolving two matrices results in a matrix. The $N_{1}$ filtered outputs are combined into a $\left(F\times T\times N_{1}\right)$ cuboid feature map after the biases are added and passed through the ReLU activation function. Cuboid convolution filters are selected in the second layer to extract more complex cross-coupled features across the cuboid feature map. The cuboid convolutions result in matrices which are combined into cuboids after adding biases and passing through ReLU activation. The cuboid is passed through the max pooling layer, flattened, and passed through the FCN. The output of the FCN of the unichannel classifier of channel d is the posterior class probability vector $P_{d}=(P_{d,\omega_{1}},P_{d,\omega_{2}},..,P_{d,\omega_{\Omega }})$, and the test input is assigned to class $\omega_{i}$ if

(5) $$P_{d,\omega_{i}}>P_{d,\omega_{j}}\;\mathrm{for}\;\mathrm{all}\;j\neq i.$$

#### 2.2.3. V-MatrixNet

The V-scalogram was introduced to overcome the inclusion of zeroed-out coefficients in the Z-scalogram. However, the resulting non-rectangular matrix presents problems for CNN classifier development because the inputs to each convolution and pooling layer are expected to be a rectangular matrix or a rectangular cuboid. The approach developed in [1] circumvents this problem by concatenating the rows of the V-scalogram, which contain only the accurately computed coefficients inside the COI, into a feature vector. The dimension of the resulting frequency-time feature vector is given by $N_{V}=\sum_{f=0}^{F-1}N_{f}$ where $N_{f}$ is the duration of the frequency band f inside the COI. The feature vector extracted from the V-scalogram $G_{V,dj}^{\left(q\right)}\left(f,t\right)$ of channel d is given by

(6) $${G_{V,dj}^{\left(q\right)}={[\nabla }_{f=0}^{\left(F-1\right)}G_{V,dj}^{\left(q\right)}\left(f,:\right)]}^{T}.$$

where ∇ represents the row concatenation operation, T is the transpose operation, and $G_{V,dj}^{\left(q\right)}\left(f,:\right)$ denotes the row f of $G_{V,dj}^{\left(q\right)}\left(f,t\right)$. The subscript j indicates that the V-scalograms are generated from the class $\omega_{j}$ m-ERPs.

**Figure 3:**
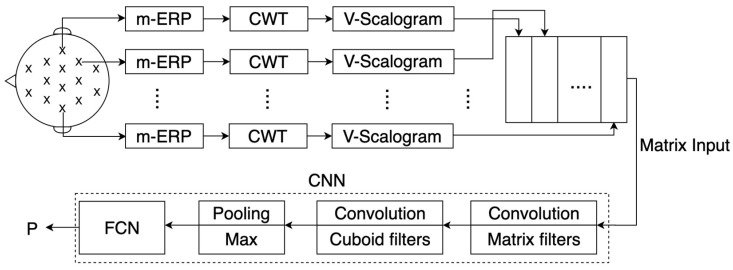
Block Diagram of the V-MatrixNet illustrating the formation of the fusion matrix and the CNN, which accepts the matrix as the input.

The V-MatrixNet, illustrated in Figure 3, fuses the V-scalogram frequency-time feature vectors of the D channels into the columns of an $N_{V}\times D$ input matrix which is given by

(7) $$G_{V,j}^{\left(q\right)}\left(f_{t},d\right)={∆}_{d=0}^{\left(D-1\right)}G_{V,dj}^{\left(q\right)},f_{t}=0,1,\ldots ,(N_{V}-1);\;d=0,1,\ldots ,(D-1).$$

where ∆ in this case is the operator for fusing column vectors into a matrix and $f_{t}$ is the column index. The index $f_{t}$ indicates that each element of the feature vector is a frequency-time element of the V-scalogram $G_{V,dj}^{\left(q\right)}\left(f,t\right)$.

The matrix $G_{V,j}^{\left(q\right)}\left(f_{t},d\right)$ can be regarded as a frequency-time($f_{t}$)-spatial(d) matrix. In the first layer, $G_{V,j}^{\left(q\right)}\left(f_{t},d\right)$ is convolved with a matrix filter $f^{\left[1,n\right]}\left(f_{t},d\right),f_{t}=-\alpha ,\ldots ,0,\ldots ,\alpha ;\;d=-\beta ,\ldots ,0,\ldots ,\beta ,n=1,2,\ldots ,N_{1}$; the output of filter n is given by the matrix convolution

(8) $$\begin{array}{c} Y_{V,j}^{\left(q\right)\left[1,n\right]}\left(f_{t},d\right)=\sum_{r=-\alpha }^{\alpha }\sum_{u=-\beta }^{\beta }f^{\left[1,n\right]}\left(r,q\right)G_{V,j}^{\left(q\right)}\left(f_{t}+r,d+u\right),\\ f_{t}=0,1,\ldots ,(N_{V}-1);\;d=0,1,\ldots ,(D-1). \end{array}$$

A bias is added to the filtered output and passed through the ReLU activation function to generate the matrix feature map. The $N_{1}$ filtered outputs are combined into a $\left(N_{v}\times D\times N_{1}\right)$ feature cuboid. The feature cuboid is convolved with cuboid filters, and the resulting feature maps are combined into a more complex feature cuboid which is passed through the pooling layer, flattened, and passed through the FCN. The softmax layer computes the posterior class probabilities, which are stored in a probability vector, and the input is assigned to the class given by the rule in Equation (3).

#### 2.2.4. V-VectorNet

The V-MatrixNet reduces to the V-VectorNet for unichannel classification. In order to determine the best channel classifier and to compare the performance against the multichannel V-MatrixNet fusion classifier, a V-VectorNet classifier is developed independently for the m-ERPs of each channel. In the first convolution layer, the column feature vector $G_{V,dj}^{\left(q\right)}$ of channel d is convolved with column filters $f^{\left[1,n\right]}\left(f_{t}\right),f_{t}=-\alpha ,\ldots ,0,\ldots ,\alpha ;\;n=1,2,\ldots ,N_{1},$ and the column output of the $n^{th}$filter is given by

(9) $$\begin{array}{c} Y_{V,j}^{\left(q\right)\left[1,n\right]}\left(f_{t}\right)=\sum_{r=-\alpha }^{\alpha }{f^{\left[1,n\right]}\left(r\right)G}_{V,dj}^{\left(q\right)}\left(f_{t}+r\right),\\ f_{t}=0,1,\ldots ,\left(N_{V}-1\right). \end{array}$$

A bias is added to the filtered output, which is a column vector, and passed through the ReLU activation function to yield the column feature map. The $N_{1}$ column feature maps are combined into a ${(N_{V}\times N}_{1})$ matrix that goes through the second convolution filter consisting of matrix filters. The matrix outputs are combined into a cuboid and passed through the max pooling layer. The output of the max pooled layer is flattened and fed into the FCN, which outputs the posterior class probability vector. The rule in Equation (5) is used to assign the input to a class.

In summary, the outputs of the convolution layers take on different shapes depending on the shapes of the inputs and the choice of convolution features. The desired fusion of the frequency, time, and spatial domains is controlled by the shapes and sizes of the filters. More complex discriminatory features can be extracted by making the networks deeper; that is, by increasing the number of network layers.

### 2.3. ERP Data Set

The EEG/ERP data used in this study to demonstrate the application and evaluate the performance of the multidomain models was the same as used in [1]. The data was downloaded from: https://eeglab.org/tutorials/10_Group_analysis/study_creation.html#description-of-the-5-subject-experiment-tutorial-data (accessed on 1 October 2022).

This data set was selected because it is compact and serves the purpose of demonstrating the application of the multidomain models for both single-subject-customized and group-based classification. Furthermore, given that the multidomain CNN models are extensions of the unichannel CNN models in [1], the performance trends can be compared to determine consistencies and/or discrepancies between the two studies. The design of the multidomain models, however, is not restricted to this particular data set or to any other data set. Details of the data relevant to this study are as follows:

Task: Auditory binary semantic task. Subjects distinguished between synonymous and non-synonymous word pairs.
Number of ERP classes: Two (synonymous, non-synonymous).
Number of subjects: 5.
Number of channels: 64.
Sampling rate: 200 Hz; Single trial duration: 1 s; Number of samples in single trials: 200
Number of single trials for each subject: 195 synonymous and 195 non-synonymous.

Complete details of the EEG data can be found on the referenced website, and the details of the m-ERPs extracted from the EEG can be found in [1].

### 2.4. Group-Based Cross-Validation

The goal of group-based cross-validation is to train the multidomain classifiers with the m-ERPs of a set of subjects and test the m-ERPs of the individual subjects not included in the training set. In order to do so in a systematic fashion, k-fold cross-validation is modified so that the folds are defined with respect to subjects. In this cross-validation approach, which we refer to as “k-subject-fold cross-validation,” each fold consists of the m-ERPs of (B/k) subjects, where B and k are the number of subjects and folds, respectively. The classifier is trained with the m-ERPs in (k−1) folds and validated (tested) on the m-ERPs of each subject in the left-out fold. As in regular k-fold cross-validation, the process is repeated k times so that the ERPs of all subjects are tested. The final result is obtained by averaging the classification accuracies within and across the k repetitions. The process can be repeated several times and averaged by first shuffling the order of the subjects so that the subjects fall in different folds. For the special case (k=B), that is, each fold contains the m-ERPs of only one subject, the procedure reduces to leave-one-subject-out cross-validation.

### 2.5. Channel Selection

Although fusing ERPs from multiple channels is likely to improve performance, designing multichannel ERP fusion classifiers is a challenging problem because the following issues have to be addressed:

(a) Should all channels be used in the design? The answer is generally no because including channels that elicit ERPs that do not carry useful discriminatory information is equivalent to adding noise, and the additional dimensions lead to overfitting, which has a negative impact on performance.
(b) If all channels are not used, how should a subset of channels be selected? Temporospatial PCA is one method that can be used to identify channels and time windows that capture effects elicited by stimuli [23,24]. Alternatively, channel selection algorithms can be applied to select a subset of useful channels [1,25,26,27].
(c) How are the ERPs of the selected channels fused? This issue has already been addressed in the development of the Z-CuboidNet and V-MatrixNet models in Section 2.2.

In general, the answers to the questions raised above are not straightforward and are often application dependent. The generalized rank-of-rank sum channel selection strategy described in [1] was used to select the top 12 channels for each subject and across all 5 subjects. The ranking for the 5 subjects pooled into a group is obtained by summing the single subject ranks and ranking the rank-sums. The selected sets of channels are listed in Table 1, in which rank 1 is the best channel and rank 12 is the 12th best channel. Observe that the channel rankings vary across the 5 subjects.

### 2.6. Morlet Wavelet Transform

The analytic Morlet CWT [1,28,29] is selected in this study because it is a good choice for analyzing the oscillatory behavior of ERPs and EEGs. The analytic Morlet mother wavelet, a product of a complex exponential signal of frequency $f_{0}$ and a zero-mean Gaussian window with variance $\sigma^{2}$, is given by

(10) $$\Psi_{f_{0}}(t)=\left(A\right){(e}^{-\frac{t^{2}}{2\sigma^{2}}})(e^{j\left(2\pi f_{0}\right)t})$$

where, the constant A ensures that the wavelet energy is equal to one. The analytic wavelet coefficients are complex, and the scalogram, represented by $G\left(f,t\right)$, is a plot of the CWT amplitude $\left|G\left(f,t\right)\right|$ or power ${\left|G\left(f,t\right)\right|}^{2}$ as a function of discrete frequency and discrete time. Scalograms of other CWTs, such as Morse [30] and Bump [31], can also be used for the development of the Z-CuboidNet and V-MatrixNet models if they are more suited for a given application.

### 2.7. CNN Architectures and Hyperparameters

The reason for selecting CNNs was explained in the Introduction. For consistency, the CNNs used in all 4 classification models had 2 convolution layers followed by a max pooling layer and an FCN. This section describes the shape, dimensions, and number of filters in the convolution layers; dimensions and strides of the max pooling filters; the number of layers in the FCN and the activations in the FCN; and the training hyperparameters.

The scalogram dimensions of each m-ERP were (f=108)×(t=200). The top D out of the 64 channels were selected; therefore, the dimensions of the input to the Z-CuboidNet models were 108×200×D. The input to Z-MatrixNet had dimensions 108×200. The feature vector extracted from the V-scalogram was a ${(N}_{V}=17,056)$-dimensional column vector (see Section 2.3); therefore, the input dimension for V-MatrixNet was 17,056×D. The input to the V-VectorNet was a 17,056-dimensional column vector. The number of filters in the first and second convolution layers are denoted by $N_{1}$ and $N_{2}$, respectively. The FCN had 3 layers of neurons. The architectures, hyperparameters, and training options of the 4 classification models are summarized in Table 2. 

## 3. Experiments and Results

This section describes the extensive set of experiments designed to evaluate the performances of the four classification models using the binary ERP data described in Section 2.3. The experimental hyperparameters were set as follows:

(a) The top D= 4, 8, and 12 ranked channels listed in Table 1 were selected to implement the multidomain classifiers in order to demonstrate the improvements that can be expected by increasing the number of channels. The unichannel classifiers were also implemented for the top 12 channels. The unichannel classifier that gave the best result, denoted by D= 1, was used for comparisons against the multidomain classifiers.
(b) Subsample averaging parameter m was set to 1, 2, and 4 to demonstrate single trial and small-average m-ERP classification.
(c) The number of single trials for each class was 195. The m-subsample averaging procedure was used to generate an equal number of m=2 and 4 m-ERPs. That is, 195 2-ERPs/class and 195 4-ERPs/class were generated.
(d) 5-fold cross-validation was used for the single-subject-customized classifier design.
(e) Leave-one-subject-out cross-validation was used for group-based classifier design.
(f) Cross-validation was repeated 50 times (50 runs). Each run began by initializing the models with a different set of random weights.

The number of m-ERPs tested for each subject was (195 m-ERPs/class) (2 classes) (50 runs) = 19,500. The classification accuracy, expressed as a percentage, was estimated as the number of correctly classified m-ERPs divided by 19,500. For group-based classification, the classification accuracy was estimated in the same manner because leave-one-subject-out cross-validation was used. That is, the number of m-ERPs in the cross-validation folds was the same for both cases. The classifiers were implemented using the PyTorch library.

### 3.1. Customized Classification Experiments

This set of experiments involved the classification of the ERPs of each individual subject using only the ERPs collected from the individual. As noted in the Introduction, the need for this approach using single trials can typically arise in the design of systems such as customized BCIs for individuals [32,33,34,35]. In order to demonstrate the improvements that can be expected by increasing the averaging parameter m, the experiments were also conducted with 2-ERPs and 4-ERPs.

#### 3.1.1. Customized Unichannel Experiments

For each subject, the Z-MatrixNet and V-VectorNet unichannel classifiers were implemented for their 12 top-ranked channels. The complete set of results is presented in Table A1, Table A2 and Table A3 in Appendix A for m taking values 1, 2, and 4. The channels labeled 1–12 in the first column of the three tables are the ranked channels listed in Table 1. For example, the results for the channel labeled CH = 1 contain the classification accuracies of the top-ranked (rank 1) channels P3, PO4, O2, O2, and C2 of Subjects $B_{1}$, $B_{2}$, $B_{3}$, $B_{4}$, and $B_{5}$, respectively. 

#### 3.1.2. Customized Multichannel Experiments

**Figure 4:**
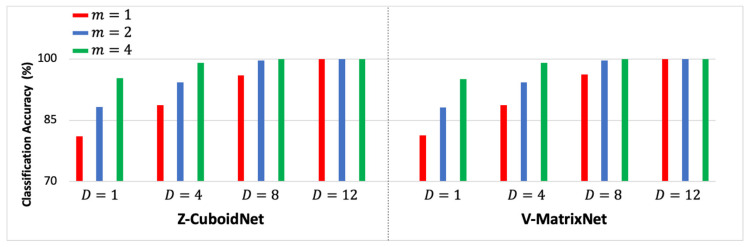
The average classification accuracies of the customized classifiers of the 5 subjects.

The Z-CuboidNet and V-MatrixNet models were implemented for the D= 4, 8, and 12 top-ranked channels listed in Table 1. The classification accuracies for each D were determined for m = 1, 2, and 4. The results are presented in Table 3. Each result in the table is the average of testing 19,500 m-ERPs. Note that m= 2 and 4 are not included for D= 12 because the multidomain models gave 100% for m=1 across all five subjects; therefore, there was no need to increase m beyond unity. The table also contains results for D=1, which is the best unichannel result for each subject extracted from Table A1, Table A2 and Table A3 in Appendix A. That is, the results for D=1 are the best Z-MatrixNet and V-VectorNet results. In order to facilitate analyses of the performance trends, the average classification accuracies of the customized classifiers of the five subjects are presented in Figure 4 as functions of D and m. That is, each bar is the average across the five customized classifiers for a given combination of D and m.

### 3.2. Group-Based Classification Experiments

**Figure 5:**
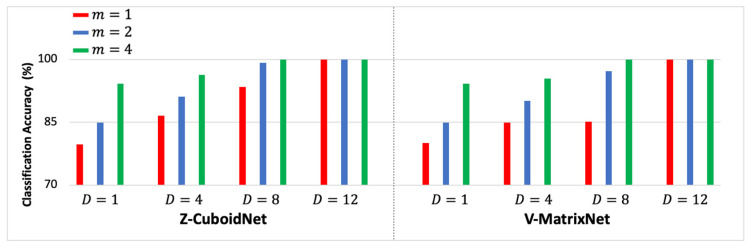
The average classification accuracies of the group-based classifiers of the 5 subjects.

The group-based classification experiments are different from the customized set of experiments in the previous section because these experiments involve classifying the m-ERPs of each subject using the models trained with the m-ERPs of the other subjects in the group. In this set of experiments, the leave-one-subject-out method was used to design the models. For example, the models were trained with the m-ERPs of subjects $B_{2}$, $B_{3}$, $B_{4}$, and $B_{5}$ and were tested on the m-ERPs of subject $B_{1}$. The results are summarized in Table 4 and are interpreted in the same manner as the results in Table 3. The 12 unichannel results are presented in Table A4, Table A5 and Table A6 in Appendix A. Unlike the customized case where the channels of each subject were ranked independently, the 12 top channels of the group-based classifiers have a common ranking. The average classification accuracies of the group-based classifiers of the five subjects are presented in Figure 5.

## 4. Discussion of Results

The four classification models can be ranked according to the classification accuracies in Table 3 and Table 4. The results in:

(a) Table 3 show that only marginal differences exist between the performances of the customized Z-CuboidNet and the V-MatrixNet classifiers across all five subjects, all values of D, and all values of m. The Z-CuboidNet and V-MatrixNet multichannel fusion models outperform the best unichannel (D=1) Z-MatrixNet and V-VectorNet models, respectively. For a given D, the performance improves by increasing m, and for a given m, the performance improves by increasing D.
(b) Table A1, Table A2 and Table A3 show that there is no single channel that is best across the five subjects.
(c) Table 4 show that the performance trends of the group-based classifiers are similar to those of the customized classifiers in Table 3. That is, the Z-CuboidNet and V-MatrixNet performances differ marginally, the multichannel multidomain models outperform the best unichannel models, and the performance improves when m and D are increased.
(d) Table A4, Table A5 and Table A6 show that there is no single channel that is best across the five subjects.
(e) Table 4 show that the accuracies of the group-based classifiers are slightly lower than those of the corresponding customized classifiers (Table 3) for small values of D and m. The drop in accuracies can be attributed to the fact that none of the m-ERPs of a test subject are included in the group training set.

Both customized multidomain models yielded single trial classification accuracies exceeding 90% for eight channels across all five subjects. Classification accuracies of 100% were obtained for all subjects when 12 channels were used. The group-based Z-CuboidNet models yielded single trial classification accuracies exceeding 90% for eight channels and yielded 100% for twelve channels across all five subjects. The single trial (m=1) average classification accuracy of the five subjects improved from 81.06 to 100% when the number of channels (D) was increased from one to twelve. This improvement is dramatic. Moreover, the ability to obtain higher accuracies by increasing the number of channels is especially noteworthy because it is a clear indication that the ERPs of the spatially distributed channels carry complementary information that can be exploited to improve performance.

There is no clear winner between the Z-CuboidNet and V-MatrixNet models for both customized and group-based classification. The zeroed-out region in the input cuboid of the Z-CuboidNet is common to all ERP classes; therefore, it is likely to be ignored by the convolution filters in the process of extracting discriminatory features during training. The Z-CuboidNet offers the most direct way of fusing the scalograms. In the previous study involving unichannel customized m-ERP classification, the results for the CNN implementations of the classifiers were mixed with no clear winner between the V-scalogram and Z-scalogram classifiers. The results of the extensions of the unichannel models to multichannel models are, therefore, in agreement with the previous study. It was also established in [1] that the V- and Z-scalogram CNN classifiers outperform the V- and Z-scalogram implementations of the other classifiers (SVM, RF, k-NN, and MLP). Undoubtedly, the Z-CuboidNet and V-MatrixNet would outperform the multichannel implementations of these other classifiers. Furthermore, the other classifiers do not offer any elegant manner to fuse the V- and Z-scalograms because they require vector inputs. Vector inputs can be generated by concatenating the rows of the scalogram of each channel into a unichannel vector, followed by the concatenation of the unichannel vectors of the multiple channels into a super-sized multichannel vector. The resulting multichannel vector input will have spatial components separated by the length of the unichannel vector, which makes it difficult to design convolution filters to locally couple the spatial information. 

Finally, it is important to note that high classification accuracies were obtained with no attempts to optimize the performance of the multidomain classifiers. In general, the multidomain classifiers have the potential for further improvements in performance by increasing the number of channels, increasing the network depth, tweaking the hyperparameters, and, when applicable, by increasing the averaging parameter m. Increasing the network depth, however, requires an increase in the training set size which could be a limitation in practical applications in which collecting large ERP ensembles is problematic. Additionally noteworthy is that although the primary focus was on ERP classification, the generalized formulations of the two multidomain models make them easily adaptable to other problems involving multisensor signal classification.

## 5. Conclusions

The Z-CuboidNet and V-MatrixNet multidomain models were introduced to improve ERP classification by fusing frequency, time, and spatial information from multichannel ERP recordings. Both CNN-based models do not require expert extraction of hand-engineered features for the input, which not only facilitates classifier design but also avoids the problems associated with selecting feature sets through trial and error. An extensive set of experiments involving customized and group-based classification of ERPs were conducted to demonstrate the application and performance of the multidomain and unichannel classifiers. The results clearly showed that the multidomain classifiers consistently outperformed the unichannel classifiers. Additionally, high classification accuracies were obtained from the multidomain models trained and tested on the single trials of single subjects, which is an especially important contribution for the design of customized classifiers for BCI-type applications in which the interface is expected to be controlled by a single stimulus presentation. It was also shown that the multidomain models trained on the ERPs from a group of subjects can accurately classify ERPs of single subjects not included in the training group, which is a notable contribution for many applications related to classifying brain activity and brain disorders. The multidomain models are highly scalable to large subject groups and to more ERP classes which are crucial requirements for practical deployment. Future efforts will focus on deploying the models for real BCI applications and large group-based brain disorder classification.

## Figures and Tables

**Table 1:** The 12 top-ranked channels for each subject and for the 5 subjects combined into a group.

	Channel Rankings											
Subject	1	2	3	4	5	6	7	8	9	10	11	12
$B_{1}$	P3	P1	P5	Pz	P7	POz	PO2	PO1	PO3	TP8	PO5	P2
$B_{2}$	PO4	F3	CP5	PO2	Oz	PO6	TP7	O1	POz	Fp1	P3	P5
$B_{3}$	O2	Oz	O1	PO6	PO4	PO2	TP7	POz	P7	PO1	TP8	PO3
$B_{4}$	O2	Oz	TP7	O1	F3	F1	F5	CP5	PO6	Fp1	PO2	PO4
$B_{5}$	C2	P5	Cz	C4	P3	P7	C1	FCz	C6	FC1	FC2	TP8
Group	Cz	C2	T8	C6	C4	C3	F6	C1	C5	CP1	Fp2	F8

**Table 2:** Architectures, hyperparameters, and training options of the 4 classification models.

	Z-CuboidNet	Z-MatrixNet	V-MatrixNet	V-VectorNet
Input dimensions	108×200×D	108×200	17,056×D	17,056×1
Conv-Layer 1: Filter dimensions; number; activations	3×3×D	3×3	3×3	9×1
	$N_{1}=32$	$N_{1}=32$	$N_{1}=32$	$N_{1}=32$
	ReLU	ReLU	ReLU	ReLU
Conv-Layer 2: Filter dimensions; number; activations	3×3×32	3×3×32	3×3×32	3×3
	$N_{2}=32$	$N_{2}=32$	$N_{2}=32$	$N_{2}=32$
	ReLU	ReLU	ReLU	ReLU
Max Pool: Filter dimensions; Stride	2×2; 2	2×2; 2	2×2; 2	2×2; 2
FCN	256, 128, 2	128, 64, 2	256, 128, 2	128, 64, 2
FCN activations	tanh, tanh, soft	tanh, tanh, soft	tanh, tanh, soft	tanh, tanh, soft
Number of Epochs	50	50	50	50
Optimizer	Adam	Adam	Adam	Adam
Learning rate	0.001	0.001	0.001	0.001
Drop out probabilities	0.15	0.15	0.15	0.15

**Table 3:** Accuracies of the customized classifiers.

Model	D	m	Subject				
			$B_{1}$	$B_{2}$	$B_{3}$	$B_{4}$	$B_{5}$
Z-CuboidNet	1	1	86.73	82.65	76.19	78.78	81.26
		2	92.35	90.79	83.61	87.31	88.45
		4	97.40	95.71	94.11	95.46	95.38
	4	1	92.70	91.50	83.16	87.10	89.13
		2	95.12	95.28	91.78	94.82	94.41
		4	100	100	97.56	98.51	99.37
	8	1	100	100	95.09	94.18	90.82
		2	100	100	100	100	98.23
		4	100	100	100	100	100
	12	1	100	100	100	100	100
V-MatrixNet	1	1	85.71	83.67	76.72	78.95	81.57
		2	92.27	91.12	85.43	88.34	86.60
		4	96.25	95.93	93.34	95.55	94.32
	4	1	92.70	91.50	83.16	87.10	89.13
		2	95.12	95.28	91.78	94.82	94.41
		4	100	100	97.56	98.51	99.37
	8	1	100	100	94.55	94.91	91.78
		2	100	100	100	100	98.00
		4	100	100	100	100	100
	12	1	100	100	100	100	100

**Table 4:** Leave-one-subject-out classification accuracies.

Model	D	m	Subject				
			$B_{1}$	$B_{2}$	$B_{3}$	$B_{4}$	$B_{5}$
Z-CuboidNet	1	1	82.81	82.64	73.62	80.34	79.32
		2	86.83	85.79	81.98	85.43	84.66
		4	96.62	95.91	90.43	94.59	93.80
	4	1	91.20	89.27	81.72	85.30	85.35
		2	93.61	94.01	86.18	90.64	91.29
		4	97.23	98.07	93.38	95.97	96.68
	8	1	95.06	95.36	93.15	93.03	90.30
		2	100	100	97.48	100	98.39
		4	100	100	100	100	100
	12	1	100	100	100	100	100
V-MatrixNet	1	1	82.86	82.72	74.88	80.68	79.25
		2	86.95	85.46	81.72	85.97	84.43
		4	97.06	95.48	90.34	94.82	93.48
	4	1	90.46	89.43	80.39	83.36	80.82
		2	93.24	92.82	84.73	89.86	90.07
		4	97.51	96.65	93.13	95.09	94.65
	8	1	88.48	87.27	84.55	84.24	80.88
		2	100	94.45	98.16	98.30	95.17
		4	100	100	100	100	100
	12	1	100	100	100	100	100

## Data Availability

Publicly available data were used in this study. The data can be found here: https://eeglab.org/tutorials/10_Group_analysis/study_creation.html#description-of-the-5-subject-experiment-tutorial-data (accessed on 1 October 2022).

## Appendix A

The customized and group-based unichannel classification accuracies of the 12 top-ranked channels are presented in this appendix. The channels, Z-MatrixNet, and V-VectorNet, are represented by CH, Z-M, and V-V, respectively. Each result in the tables is the average of testing 19,500 m-ERPs. The best result across the 12 channels is in boldface.

**Table A1:** Unichannel accuracies of the customized classifiers for single trials (1-ERPs).

CH	Subject									
	$B_{1}$		$B_{2}$		$B_{3}$		$B_{4}$		$B_{5}$	
	Z-M	V-V	Z-M	V-V	Z-M	V-V	Z-M	V-V	Z-M	V-V
1	**86.73**	**85.71**	**82.65**	**83.67**	75.51	74.13	**78.78**	77.21	81.03	81.39
2	84.97	85.03	81.97	82.45	75.85	76.11	77.55	78.44	80.85	81.05
3	84.69	83.67	82.52	81.29	**76.19**	76.53	76.94	78.23	81.14	80.67
4	83.83	84.01	81.29	82.85	75.29	**76.72**	78.37	77.97	**81.26**	81.33
5	82.95	83.81	80.27	80.95	74.78	75.58	76.19	**78.95**	80.42	81.12
6	82.99	84.13	79.05	79.59	74.15	74.63	77.62	78.20	80.35	80.52
7	81.22	81.63	79.12	78.44	73.40	73.27	75.29	76.34	80.93	80.06
8	81.69	81.98	78.91	77.76	74.49	72.24	76.05	76.16	80.61	80.82
9	81.81	81.13	78.31	78.14	73.48	73.01	75.42	76.19	80.49	81.32
10	81.90	81.63	77.34	78.96	73.76	74.90	77.70	75.74	80.19	80.26
11	81.28	81.55	78.20	78.81	73.64	73.39	77.48	76.79	80.07	80.21
12	81.80	81.14	78.85	78.66	73.15	73.25	78.07	78.17	80.31	**81.57**

**Table A2:** Unichannel accuracies of the customized classifiers for 2-ERPs.

CH	Subject									
	$B_{1}$		$B_{2}$		$B_{3}$		$B_{4}$		$B_{5}$	
	Z-M	V-V	Z-M	V-V	Z-M	V-V	Z-M	V-V	Z-M	V-V
1	**92.35**	90.48	90.44	89.08	83.27	84.10	**87.31**	87.20	87.06	86.06
2	91.67	92.21	88.73	90.97	83.56	82.90	86.17	87.10	85.78	83.61
3	88.94	91.77	**90.79**	89.42	83.27	84.85	86.70	85.92	85.65	86.25
4	91.70	92.14	89.05	90.79	82.58	83.60	85.49	86.41	**88.45**	83.48
5	90.71	91.30	88.59	90.91	82.91	82.78	85.41	84.95	84.17	85.94
6	91.35	89.13	88.75	**91.12**	82.04	**85.43**	86.79	**88.34**	87.98	84.11
7	91.38	91.97	89.85	90.08	**83.61**	85.14	85.49	85.51	88.11	**86.60**
8	90.41	**92.27**	89.42	88.79	81.66	83.68	85.27	86.27	87.58	85.75
9	89.55	91.38	89.67	89.28	82.73	84.06	84.73	87.81	85.82	85.68
10	91.40	90.93	89.88	87.59	81.36	83.98	83.24	87.71	85.02	83.17
11	91.35	89.61	88.64	88.35	80.85	82.90	85.76	85.11	83.64	86.13
12	89.70	91.83	87.42	88.49	81.41	82.73	85.76	86.30	83.84	86.16

**Table A3:** Unichannel accuracies of the customized classifiers for 4-ERPs.

CH	Subject									
	$B_{1}$		$B_{2}$		$B_{3}$		$B_{4}$		$B_{5}$	
	Z-M	V-V	Z-M	V-V	Z-M	V-V	Z-M	V-V	Z-M	V-V
1	96.23	95.96	**95.71**	93.34	**94.11**	91.77	95.08	93.74	94.79	**94.32**
2	95.79	94.69	92.50	94.53	92.19	93.02	**95.46**	**95.55**	94.75	91.29
3	96.34	**96.25**	94.12	92.66	93.09	**93.34**	94.98	94.09	**95.38**	92.17
4	96.23	95.94	93.99	95.12	91.66	92.34	93.96	95.30	93.68	92.30
5	94.52	94.78	94.11	92.89	93.74	91.56	95.25	94.82	93.62	92.38
6	94.35	94.92	95.20	**95.93**	92.66	90.68	94.44	94.14	93.43	93.97
7	**97.40**	96.06	93.43	93.01	91.65	92.64	94.04	92.61	94.91	93.81
8	94.50	94.83	91.24	95.24	90.15	91.18	94.85	94.50	92.19	91.98
9	93.45	93.11	93.23	92.93	91.85	90.16	94.53	92.38	92.93	91.95
10	95.89	94.96	94.35	94.55	92.64	92.64	93.64	93.82	93.20	91.73
11	95.16	96.09	91.84	93.45	91.75	90.85	94.96	93.97	91.11	92.98
12	94.22	95.34	93.37	91.93	91.86	91.55	93.35	94.28	93.15	93.24

**Table A4:** Leave-one-subject-out unichannel accuracies for 1-ERPs.

CH	Subject									
	$B_{1}$		$B_{2}$		$B_{3}$		$B_{4}$		$B_{5}$	
	Z-M	V-V	Z-M	V-V	Z-M	V-V	Z-M	V-V	Z-M	V-V
Cz	81.43	**82.86**	81.70	**82.72**	72.52	71.44	**80.34**	**80.68**	**79.32**	**79.25**
C2	82.52	82.64	81.36	81.22	**73.62**	72.59	79.93	78.65	78.16	78.91
T8	82.28	82.50	81.79	82.21	72.51	73.13	79.66	79.32	78.70	79.01
C6	81.25	81.88	**82.64**	82.33	72.32	**74.88**	78.65	79.79	77.30	77.91
C4	81.76	82.19	80.61	81.60	72.63	73.12	79.83	79.32	77.47	77.84
C3	80.99	80.82	80.95	80.18	72.82	72.69	79.12	78.85	77.61	76.91
F6	**82.81**	82.39	80.06	81.10	72.80	73.31	79.23	79.04	78.46	77.89
C1	82.65	81.93	80.94	80.19	71.12	73.70	78.72	78.99	76.81	76.26
C5	81.51	81.99	81.45	81.13	72.06	72.56	76.76	78.92	76.67	77.41
CP1	82.19	80.93	81.09	81.53	72.56	72.93	79.80	79.66	76.80	77.01
Fp2	81.63	82.09	81.54	80.77	71.98	72.39	78.85	78.98	75.76	75.58
F8	81.58	81.86	81.17	82.13	72.19	70.30	78.57	77.96	76.36	75.45

**Table A5:** Leave-one-subject-out unichannel accuracies for 2-ERPs.

CH	Subject									
	$B_{1}$		$B_{2}$		$B_{3}$		$B_{4}$		$B_{5}$	
	Z-M	V-V	Z-M	V-V	Z-M	V-V	Z-M	V-V	Z-M	V-V
Cz	**86.83**	85.95	**85.79**	85.35	81.80	**81.72**	84.73	85.13	**84.66**	83.93
C2	85.95	**86.95**	85.33	84.36	79.79	80.81	84.51	**85.97**	83.42	84.33
T8	85.70	85.19	84.46	83.87	79.12	79.23	**85.43**	84.95	83.62	**84.43**
C6	86.66	85.13	84.31	85.31	**81.98**	79.46	85.38	85.55	83.14	83.12
C4	86.17	86.33	84.34	**85.46**	81.00	81.05	84.08	85.50	82.64	82.91
C3	86.10	86.48	85.34	85.16	80.92	78.61	85.11	85.31	82.90	83.91
F6	86.23	86.34	84.44	84.25	80.62	80.46	84.40	85.55	82.73	83.57
C1	85.57	85.32	83.85	84.17	81.44	79.17	84.63	85.50	82.92	82.27
C5	86.51	86.42	84.14	83.42	81.47	81.41	84.82	84.77	83.16	82.16
CP1	86.51	86.17	83.39	83.60	80.44	81.35	85.41	84.69	83.29	83.01
Fp2	85.40	85.15	84.73	83.94	79.50	79.59	85.00	84.08	82.44	81.88
F8	84.29	83.93	83.07	83.46	79.38	77.89	83.76	83.75	81.57	82.08

**Table A6:** Leave-one-subject-out unichannel accuracies for 4-ERPs.

CH	Subject									
	$B_{1}$		$B_{2}$		$B_{3}$		$B_{4}$		$B_{5}$	
	Z-M	V-V	Z-M	V-V	Z-M	V-V	Z-M	V-V	Z-M	V-V
Cz	95.02	95.44	94.64	93.61	90.06	**90.34**	92.77	92.20	92.82	93.36
C2	96.05	**97.06**	**95.91**	94.48	**90.43**	87.75	91.05	**94.82**	92.27	91.61
T8	95.52	96.70	93.96	92.62	89.58	87.82	**94.59**	94.80	93.76	93.35
C6	93.36	96.44	92.39	93.53	88.83	88.75	91.79	94.70	92.19	93.26
C4	93.32	93.71	92.97	92.64	90.21	89.06	92.33	94.72	91.69	**93.48**
C3	96.11	93.79	94.00	95.03	88.82	89.12	92.19	91.53	**93.80**	92.27
F6	**96.62**	93.85	91.51	**95.48**	86.57	87.31	92.70	94.41	93.51	92.72
C1	95.14	93.92	91.95	93.40	86.98	87.13	91.87	93.32	93.65	93.28
C5	93.44	94.01	92.13	94.74	89.95	88.19	93.65	92.06	93.43	92.05
CP1	96.30	95.70	93.17	93.18	88.44	88.42	90.42	91.49	92.76	92.87
Fp2	94.34	93.53	91.30	93.12	87.84	89.36	92.98	94.47	92.18	93.44
F8	94.21	95.89	90.84	94.33	86.87	89.22	92.95	92.58	91.24	92.78
